# Lessons learned from the rapid development of a statewide simulation model for predicting COVID-19’s impact on healthcare resources and capacity

**DOI:** 10.1371/journal.pone.0260310

**Published:** 2021-11-18

**Authors:** Stacy Endres-Dighe, Kasey Jones, Emily Hadley, Alexander Preiss, Caroline Kery, Marie Stoner, Susan Eversole, Sarah Rhea

**Affiliations:** 1 RTI International, Research Triangle Park, North Carolina, United States of America; 2 College of Veterinary Medicine, North Carolina State University, Raleigh, North Carolina, United States of America; Universidade de Vigo, SPAIN

## Abstract

The first case of COVID-19 was detected in North Carolina (NC) on March 3, 2020. By the end of April, the number of confirmed cases had soared to over 10,000. NC health systems faced intense strain to support surging intensive care unit admissions and avert hospital capacity and resource saturation. Forecasting techniques can be used to provide public health decision makers with reliable data needed to better prepare for and respond to public health crises. Hospitalization forecasts in particular play an important role in informing pandemic planning and resource allocation. These forecasts are only relevant, however, when they are accurate, made available quickly, and updated frequently. To support the pressing need for reliable COVID-19 data, RTI adapted a previously developed geospatially explicit healthcare facility network model to predict COVID-19’s impact on healthcare resources and capacity in NC. The model adaptation was an iterative process requiring constant evolution to meet stakeholder needs and inform epidemic progression in NC. Here we describe key steps taken, challenges faced, and lessons learned from adapting and implementing our COVID-19 model and coordinating with university, state, and federal partners to combat the COVID-19 epidemic in NC.

## Introduction

The United States is actively engaged in an effort to halt the physical and societal impacts of a deadly disease (COVID-19) caused by a novel coronavirus (SARS-CoV-2). In North Carolina (NC), the first case of COVID-19 was detected on March 3, 2020 [1]. An Emergency Task Force [2] was established but by the end of April 2020 the number of confirmed cases had soared to over 10,000. NC health systems faced intense strain to support surging intensive care unit (ICU) admissions and avert hospital capacity and resource saturation. Even advanced health systems can be stretched beyond capacity, resulting in worst-case scenarios for rationing of care [3].

Public health emergencies, such as the current pandemic, exemplify the importance of anticipating hospital resource needs [4]. An effective public health response requires leaders to make critical decisions, with little or incomplete information, in a rapidly evolving environment. Modeling can be used to provide leaders with reliable data needed to better prepare for and respond to public health crises [5].

**Fig 1:**
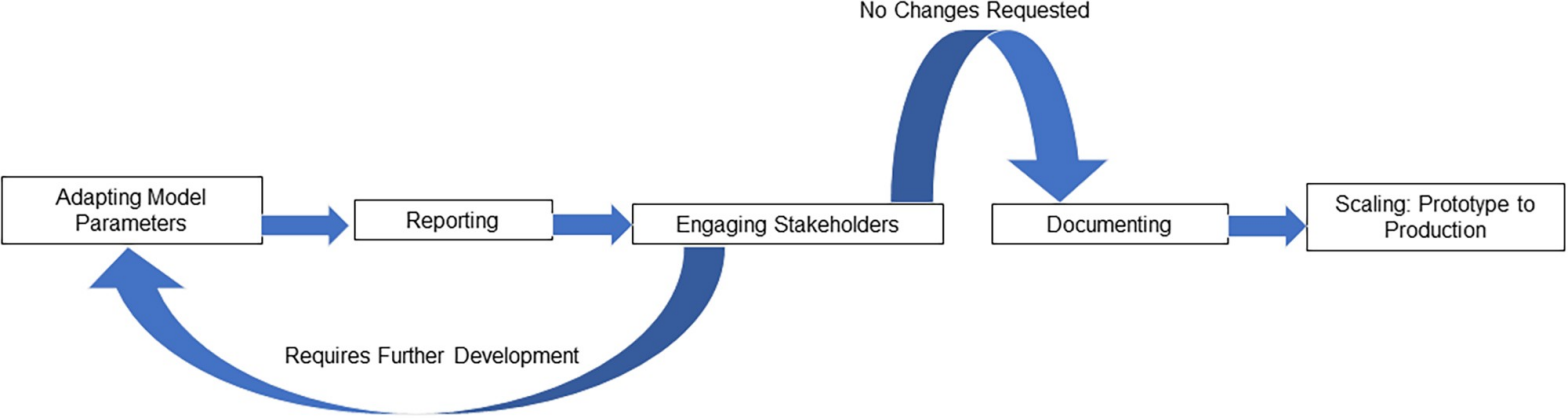
Model adaptation process.

**Fig 2:**
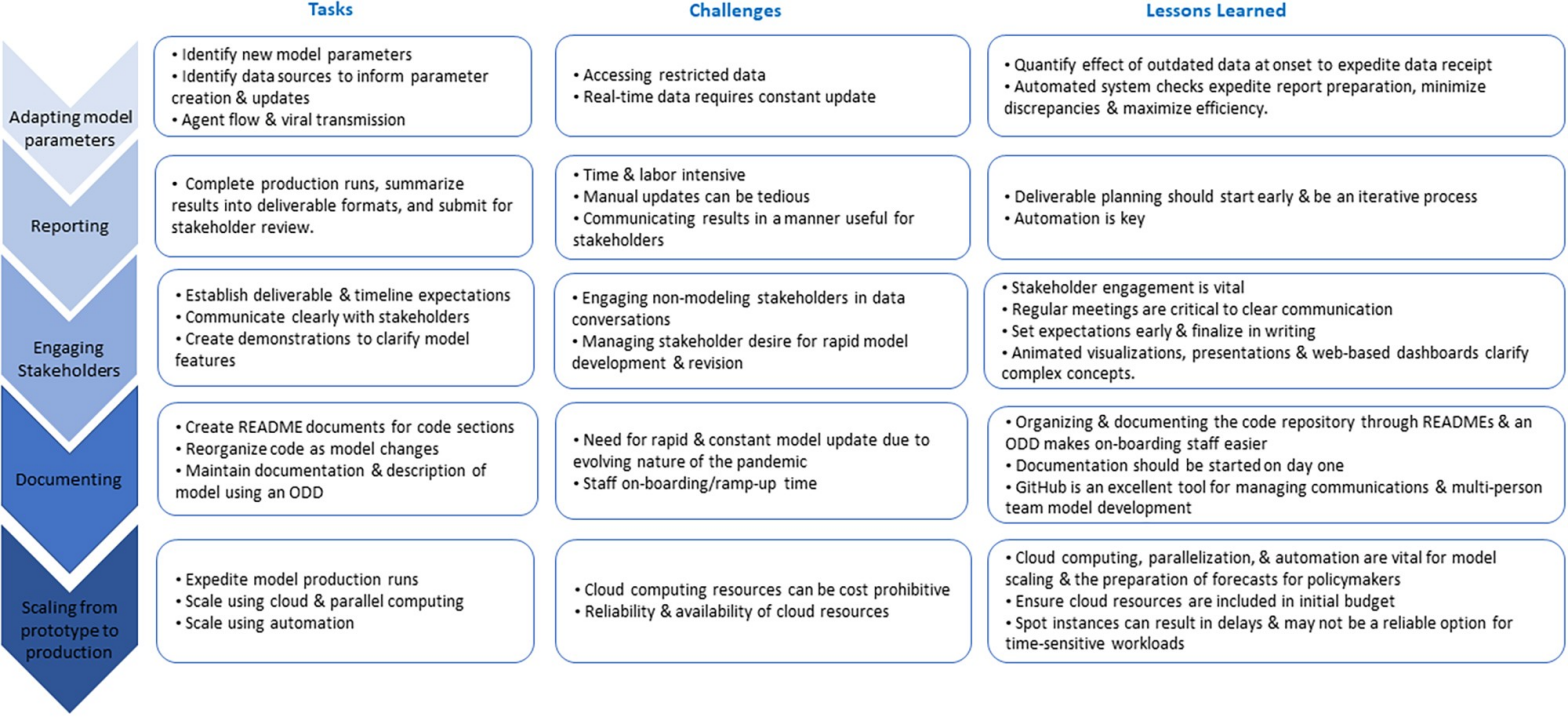
Tasks, challenges, and lessons learned from the rapid development of a statewide model for predicting COVID-19’s impact on healthcare resources and capacity.

The Centers for Disease Control and Prevention (CDC) established the “Urgent COVID-19 mathematical modeling of healthcare impact and capacity” program to predict COVID-19’s impact on U.S. healthcare facilities, resources, and capacity. RTI International has a long history of successful collaboration with CDC and NC stakeholders to model infectious diseases in NC healthcare settings [6]. To support CDC in addressing the pressing need for reliable COVID-19 forecasting data, RTI adapted a previously developed geospatially explicit healthcare facility network model to predict COVID-19’s impact on healthcare resources and capacity in NC. The model adaptation was an iterative process requiring constant evolution to meet stakeholder needs and inform epidemic progression in NC (Fig 1). The following sections describe key steps taken, challenges faced, and lessons learned from adapting and implementing our COVID-19 model and coordinating with university, state, and federal partners to combat the COVID-19 epidemic in NC (Fig 2).

## Methods

Using a geospatially explicit synthetic population, we developed an agent-based model (ABM) [7] of agent (i.e., patient) movement through NC’s healthcare systems [6, 8]. This ABM served as a framework for agent flow and had a disease-specific natural history submodel for a healthcare-associated infection. The model was designed so that submodels for different diseases could be substituted. The breadth and flexibility of the existing ABM made it an effective platform which could be adapted to forecast hospital bed demand during the COVID-19 pandemic, including detailed information about patient movement among acute care hospitals, long-term acute care hospitals, nursing homes, and the community.

During the public health response activity described here, non-public hospital census data for model initialization were provided by public health partners to the modeling team. Because these non-public data are available only through a data use agreement, for the purposes of model sharing, we used publicly available data for default hospital census values at ABM initialization [9]. The RTI Institutional Review Board reviewed this study and determined that it does not constitute research with human subjects.

### Data sources and validation

#### Data sources for model calibration

Several data sources and model parameters were used to adapt the existing ABM to simulate COVID-19 cases and subsequent hospitalizations. Transition probabilities for movement to and from hospital facility nodes in the ABM had been calibrated to hospital discharge data from 2017 [10]. During the pandemic, there was a reduction in elective procedures causing non–COVID-19-related hospitalizations to drop, reducing the accuracy of previously calculated transition probabilities. Current hospitalization counts for both ICU and non-ICU patients (without COVID-19) were needed to recalculate non–COVID-19-related hospitalization probabilities. We followed the same method for creating transition probabilities as the previous calibration [10].

Starting in September 2020, we obtained access to current hospital occupancy data on the number of patients at each hospital in NC disaggregated by patient COVID-19 status and ICU status. These data were updated once a week. Non–COVID-19-related daily hospitalization probabilities were recalculated using the new data on non–COVID-19-related hospitalizations. Hospital census data were also used to initiate the number of COVID-19 and non–COVID-19 agents at each hospital for both ICU and non-ICU beds. In addition to the hospital census data, other data sources [11–15] and parameters from the previous calibration effort [10] were used to finish calibrating agent movement. Documentation for calculating these probabilities is found in the model’s Overview, Design Concepts, and Details (ODD) protocol [9]; all code is available on the model’s public code repository [16]. New data were put through an automated checking system to make sure received data were in a consistent format and contained the same hospitals as the previous week.

To simulate hospitalizations related to COVID-19, the adapted model functioned by selecting a projected number of agents to become infected. At the time of model creation, several modeling teams were only projecting case counts for 2–4 weeks. After discussion with our stakeholders, we decided that 30-day hospitalization estimates were most appropriate. Separate compartmental models were built to create these case forecasts [9]. Using up-to-date COVID-19 case counts, 30-day estimates for COVID-19 cases by county were created using susceptible-exposed-infectious-recovered (SEIR) compartmental models. Output of the SEIR models was used as input for the ABM. These projections were then evaluated to ensure that forecasted case counts matched the expected growth of cases. If we could accurately forecast COVID-19 case counts beyond 30 days, we would have considered providing stakeholders output for a longer time horizon.

When an agent was given COVID-19 in the ABM, they were also assigned a severity level and a probability of needing hospitalization. Using an application of the Bayes equation, the probability of hospitalization given age, presence of comorbidities, and whether the agent was tested for COVID-19 were calibrated to match up-to-date data on COVID-19 hospitalizations. These parameters were tested to ensure that the overall probability for COVID-19 hospitalization stayed constant while probabilities for specific age group, comorbidity, and COVID-19 severity combinations were updated to match provided data.

#### External data sources and validity checks

Parameters from data provided by the state were prioritized. However, when needed, other parameters from external data sources were also used. Because of lack of empirical data, a weekly system to identify new parameters in recently published literature was developed and used to validate our model output against other sources. Every week, literature from the COVID-19 literature surveillance team [17], Johns Hopkins [18], and the daily email from the University of Washington was reviewed [19]. Key findings were extracted and added to a cumulative document tracking key parameters related to epidemiological characteristics (e.g., incubation period, reproductive number), hospitalizations, characteristics of infectious persons, information from other models, and any other key information. Additional data searches and reviews were also completed whenever a decision was made to update parameters.

A series of validation checks for model output were conducted weekly. First, the estimated number of hospitalizations and infections from the model was compared to forecasts from up to nine other publicly available models to confirm that model estimates were within a reasonable range. Second, model output was also compared with observed data from the state including overall hospitalizations, hospitalizations in the last 12 hours, and ICU hospitalizations for patients with COVID-19. Finally, effective reproductive number estimates, which are tied directly to case counts in the model, were compared to five publicly available models to decide which of the modeled scenarios were most likely.

### Reporting

#### Weekly deliverables for stakeholders

To facilitate rapid, informed decision-making about the SARS-CoV-2 crisis, stakeholders required model findings to be communicated clearly, concisely, and regularly. Production runs were completed weekly from June through December 2020. Each production run consisted of 100 simulations each for three R_e_ ranges: 1–1.2, 1.2–1.4, and 1.4–1.6. For each R_e_ range, the primary reported outcome was the mean and interquartile range of hospital demand over time across the 100 simulations. The results were summarized into three deliverable formats (see Table 1). The formats were a result of multiple rounds of conversation with various stakeholders who needed model output. Weekly deliverables were also developed via an iterative process with input from state and federal stakeholders. Early in the development process, the technical team generated a variety of visualizations that might be useful. Stakeholders provided feedback at regular meetings on suggestions to improve the usefulness of the deliverables.

**Table 1:** Weekly deliverables for key stakeholders.

Deliverable Description	Audience
Excel file summarizing outcome variables by region and state	Internal and external technical stakeholders
Tableau workbook with interactive visualizations	Detail-oriented stakeholders looking to interact with plots
PowerPoint slides with static versions of selected plots in the Tableau workbook	High-level stakeholders seeking quick summaries

#### Scaling

In June, a single production run (consisting of 300 model runs) would take over 20 hours to complete. After a production run was finished, an additional 4 hours of manual work was required to generate files for the weekly deliverable. If a change to input parameters was requested, a server error occurred, or a code bug was found, the entire process would need to be restarted. As model runs and weekly deliverables grew in complexity, it quickly became apparent that adaptations were needed.

Scaling efforts included cloud computing, parallel computing, and automation. These efforts all shared the same goal: to reduce the amount of time needed to provide output to stakeholders. The use of cloud computing doubled the available computing power. To reduce costs, Amazon Web Services Spot Instances [20] were used instead of on-demand cloud resources. Spot Instances offered access to idle cloud resources at a reduced cost, with the caveat that the service can be interrupted if the resources are needed for on-demand users. GNU Parallel [21] was used to parallelize simulations in a production run across computing resources. Parallel computing permitted multiple model runs to be completed in tandem, saving time and resources.

Finally, Python and shell scripts were written to automate as many of the steps involved in a production run as possible. Docker containers were used to deploy and run the Python models on cloud servers, ensuring that the models would run as expected and without package installation issues. By the end of the project, a single script was able to access cloud resources, create and execute the required 300 simulations, gather and aggregate all the results, and remove all data from the cloud resources. Additionally, automation was especially helpful when it came to creating weekly deliverables. The following automation steps were implemented to reduce the manual workload:

- Full automation of the Excel file, including automated generation of a data dictionary, using the xlwings Python package.
- Partial automation of the Tableau workbook, including automation of the underlying data file and reuse of a Tableau workbook template each week.
- Full automation of the creation of the PowerPoints using the python-pptx Python package.

Automation reduced the time it took to generate weekly deliverables to just a few minutes, with minimal additional time needed for manual review.

Through these improvements we were able to reduce model runtime from 20 hours to 15 minutes, and deliverable generation from over 4 hours to minutes. Including a multistage validation and review process, deliverables could be turned around 1 business day after receiving updated input data.

#### ABM animation

**Fig 3:**
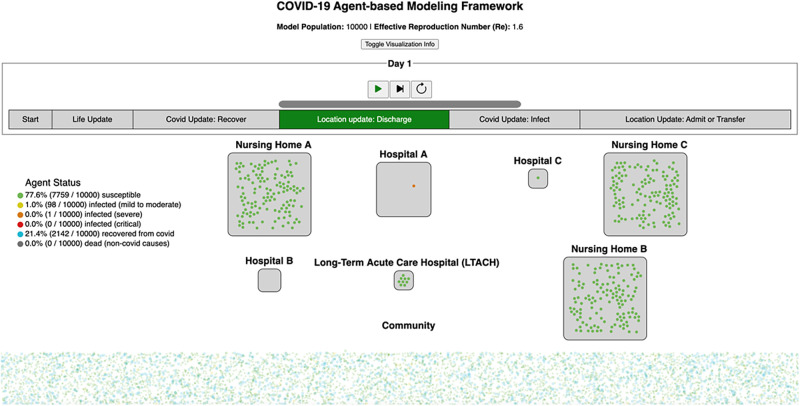
Screenshot of the COVID-19 ABM animation at hospital discharge.

Model updates were explained during weekly stakeholder meetings. Animated visualizations (Fig 3) were used to introduce basic concepts of the ABM and improve stakeholder interpretation of model output. Animations served to provide visual demonstration of agent movement through locations and disease states. The animation also depicted the steps and assumptions made by the ABM allowing for improved communication with stakeholders.

### Code and documentation

#### Code repository

Git, a file repository system commonly used with open-source code, was used to facilitate collaboration and communication among the technical contributors. GitHub, a platform for Git, was used for code storage and version control, enabling multiple users to contribute simultaneously and the project to maintain multiple versions of the codebase. Separate GitHub repositories were maintained for model code and for code to generate weekly deliverables. Using GitHub allowed the project to maintain a clean, stable version of the model to use in the weekly runs, and a version which had all the current updates under development.

Our ABM code required constant evolution to meet stakeholder needs and inform epidemic progression in NC. To accommodate this need, the code repository was continually reorganized and consolidated as model updates were made. Automated tests were added to ensure that key code functionality was not affected by updates. Documentation was added to each major section of the code repository in the form of README files, a common file format for explaining the contents of a directory in a repository. Updating the affected READMEs was considered an integral part of the code review process, and changes were not allowed unless the necessary documentation was updated.

#### Overview, design concepts, and details protocol

The accompanying README files for the code repository were utilized by the modeling technical staff working on the model runs and reports. Stakeholders and even other nonmodelers on the project team did not routinely access these documents. Instead, an ODD protocol was maintained and shared regularly with the full project team and stakeholders. This document is a commonly accepted method for describing an ABM and is usually a supplement document for journal articles written about the model [9].

For this project, an up-to-date ODD protocol was maintained throughout. Markdown files, a formatted text file common in repositories, were used to separate the ODD protocol into nine parts: one for the introduction, one for the appendix, and one for each of the seven main sections of an ODD protocol. We decided to use Markdown so that our documentation could live on our team’s repository and so we could make use of Git to store earlier versions of the documentation. The goal was that all versions of the model would be accompanied by an updated ODD protocol. To do this, all code and data changes were required to be accompanied by corresponding changes to the ODD protocol. When sharing the ODD protocol with nontechnical staff or a nontechnical audience, the Markdown files were exported to a single PDF [9].

### Project management and partner/stakeholder relations

From its onset, the project was in a constant state of evolution and expansion. It quickly became apparent that more technical staff were required to meet stakeholder needs for urgent, reliable data to inform response to the COVID-19 crisis. Visualizing model results also became critical as the state’s needs for communication of predictive modeling grew. This required that staffing on the project be shifted to include additional visualization specialists, especially as retrospective analysis of results provided to the state over time were analyzed for both effectiveness and accuracy. Staff onboarding had to be expedited to keep pace with the rapidly evolving pandemic and urgent need for evidence-based data to inform policy and decision making. Onboarding new staff required clear, standardized communication. Several tools were used for streamlining the process including an organizational chart, the ODD protocol, Roles and Responsibilities document, Quality Management Plans, and regular internal Project Review Meetings. Regular meetings, both internal and with state and federal stakeholders, were also held and served to keep parties abreast of model updates and to inform changes to the delivery of model results.

## Results and lessons learned

### Data sources and validation

Obtaining access to hospital data relevant to model development was critical. We facilitated communication of the importance of the hospital data to stakeholders by quantifying the problems caused by using outdated datasets, specifically outdated hospital bed counts and yearly hospitalization totals. Hospitalization probabilities had originally been calibrated to data from 2017. Not only had hospitals changed in size since 2017, but new hospitals had been built and old hospitals had been closed.

Real-time data are messy, especially when those data rely on several reporting systems. For the first reports created using the hospital occupancy data, hospital names were manually checked, as was the last date each hospital reported occupancy. The number of reporting hospitals, the names of these hospitals, and the number of days that they reported occupancy could change over time. If data being received are constantly changing, it is vital that an automated checking system be put into place immediately. We recommend writing tests that can catch and identify issues before they cause issues later in the model pipeline.

Most COVID-19 model parameters which were employed were point estimates (e.g., *R*_e_ value, starting hospitalization totals, COVID-19 hospitalization rate). Future model activities could prioritize use of parameter distributions and facilitate ease of completing sensitivity analyses to help stakeholders answer what-if questions.

Over the course of model development, we sought to balance updating the model with new parameter values or assumptions with communicating those changes to stakeholders. We developed a system whereby we would first discuss any potential changes with stakeholders and then develop documentation, with references about why updates were needed and the source of the new parameters. Next, we would examine the impact of the parameters on model estimates and come to a joint conclusion about what updates were necessary and appropriate. This process highlighted the importance of getting buy-in from stakeholders about parameters and model adjustments while balancing the impact of those parameters/updates on the model output.

### Reporting

#### Weekly deliverables

Deliverables are a crucial but potentially overlooked aspect of model development—a great model is only useful when the results can be clearly communicated with stakeholders. This project used three types of weekly deliverables, as described above, to reflect the varying needs of stakeholders. These needs were determined through regular discussions in the model development phase and reflect the lessons learned that deliverable planning should start early and is an iterative process.

One key insight was determining what the y-axis would be for the PowerPoint deliverables described in Table 1. These slides were often shared widely and looked at briefly by many stakeholders, so it was important that they were easy to interpret quickly. Early versions used a y-axis that estimated the number of beds above current capacity that were needed. However, stakeholders informed us that the total bed count needed would be much more useful. The PowerPoint deliverable was updated to reflect this stakeholder need.

Automating the generation of weekly deliverables was a key lesson learned that had several benefits. Through these improvements we were able to reduce model runtime from 20 hours to 15 minutes and deliverable generation from over 4 hours to minutes. Including a multistage validation and review process, deliverables could be turned around 1 business day after receiving updated input data. One benefit of automation was the considerable reduction in labor time, which allowed the analyst more time and energy to review the deliverables for validity and quality rather than focusing on generating the deliverables themselves. Other benefits were reducing opportunities for an analyst to make mistakes and ensuring consistency between weekly deliverables because the same code was used every week. Although time and labor were needed for the initial development of the deliverable automation process, the investment in automation allowed for fast, high-quality, consistent weekly deliverables.

#### Scaling

Cloud computing, parallel computing, and automation were vital to scale our model and provide timely forecasts to policymakers. However, they also presented several challenges. For example, all these steps required additional planning and development time. We had to build complex infrastructure to parallelize model runs and take advantage of cloud computing resources. To build this infrastructure, we also had to onboard additional software developers to the project. Much of this complexity stemmed from our use of local servers (with no cost to the project) alongside cloud resources. Simulation jobs had to be distributed across multiple servers. Although this reduced cost, it may not have been worth the added complexity. Future projects will explore whether using a single cloud resource powerful enough for the workload offers a better balance of computing cost and development cost. Similarly, automation eventually saved our team a huge amount of time but required a large initial time investment.

Cloud computing resources also led to two additional challenges: funding and reliability. Cloud resources became an ongoing cost that was not included in the initial budget. Spot instances were used instead of on-demand instances to minimize this unexpected cost but this led to a reliability issue. Spot instances are not always available and are, therefore, ideal for fault-tolerant, flexible workloads. Spot instances are not, however, well suited for time-sensitive workloads like our weekly production runs. Unavailable spot instances can lead to delays and added stress.

Automation was crucial to reducing the number of manual steps in a model run and making weekly reporting feasible. However, the time we invested attempting to automate certain, more fragile steps ended up being wasted effort. Generating *R*_e_ estimates required the analyst to pay close attention to warning messages that may arise while estimates are being generated, such as divergent transitions in the Hamiltonian Monte Carlo algorithm underlying our chosen estimation method. The initial effort to automate this step did not keep the analyst sufficiently in the loop to handle such warnings. To fully automate this process, we would have had to build complex log monitoring logic to handle all possible warnings and responses. Ultimately, we decided it was more efficient to run this step manually.

#### ABM animation

The ABM visualization was a helpful tool for communicating how the model worked to stakeholders who did not have a background in modeling. However, it was not developed until late in the project. The feedback was strongly positive when the tool was presented, and some stakeholders stated that the visual representation allowed them to understand the model in ways that earlier explanations verbally, in writing, and through presentations had not.

### Documentation and code

#### Code repository organization

GitHub was useful in several ways. The GitHub *wiki* feature was particularly helpful because it gave contributors a place to store internal documentation. This allowed contributors to write READMEs in a way that could be made public while keeping private documentation separate. Because it is written in Markdown, the *wiki* was also easy to edit. The GitHub *issues* and *branching* features were useful for managing communications and development on a multi-person team. It was also easier to facilitate publishing the code publicly because all model programming had been done in GitHub throughout the project with an understanding that the work would someday be public. However, we found that a backlog of unresolved GitHub issues could quickly build up because of the rapid nature of development. Regular pruning (removing of outdated issues) of the backlog and reviewing the backlog with members outside of the technical team helped to mitigate this backlog.

The value of organizing and documenting the repository early could not be overstated. The code for the ABM evolved from a repository devoted to modeling healthcare-associated infections. Early on, many changes had to be made quickly to accommodate the adaptation of the model to COVID-19. This caused an already-complicated codebase to evolve rapidly as ad hoc changes were applied to address each new request. By requiring a review of the changes to be conducted by a technical team member who did not make the changes, several bugs and code errors were avoided. The decision to maintain a separate repository for reporting ensured that we could maintain a clear separation between updates that required model changes and requests for changes in output reporting. This was helpful as the reporting requests increased in scope and specificity.

While the codebase was expanding in complexity, the team was also adding new members to accommodate the increase in tasks. We took several approaches to help facilitate onboarding of new team members. First, the organization and documentation of the repository was updated based on new staff feedback, simplifying wherever possible to iron out sources of confusion. Second, new team members traced over the entire codebase, taking notes on how various parts of the model initialized, updated, and interacted with each other. Finally, these efforts were augmented by a project culture that encouraged team members to ask questions and to initiate team discussions when questions got too complicated. By taking the time for these efforts, we not only made it easier for new team members to learn the project, but also uncovered new bugs and areas of improvement in the codebase.

#### Overview, design concepts, and details protocol

Although writing an ODD protocol was always planned for this ABM, the initial ODD writeup did not start immediately. We found that the earlier in the process this document can be initiated, the greater the ease of maintaining and keeping it updated. To aid in ODD protocol development, coders were asked to update the description of any new functionality within the ODD protocol anytime they submitted changes to model code. Although this helped maintain an up-to-date ODD protocol, we had to balance that by the burden on both the reviewer of these changes and the person submitting the changes.

Finally, an ODD can be a great onboarding tool for new staff. ODDs are designed so that the model can be replicated by following the content with the ODD. New staff should read the ODD as they begin work on an ABM that is in development.

### Project management and partner/stakeholder relations

Keeping pace with ever-evolving pandemic-related data is an immense challenge. Our project and staff had to be innovative, agile, and adaptable to accommodate this rapid evolution. Clear lines of communication and standardized instruction were one of the key pillars in the success of this project. Regularly scheduled meetings worked well and served as a mechanism to keep modelers abreast of the many changes and to do a pulse-check on overall staff stress levels. Task leader meetings were helpful to strategize tasks and ensure that the team and leadership were in line about expectations. Despite the recognized benefit of weekly stakeholder meetings, state personnel were essential to the pandemic response, which severely limited their availability. The challenge of time constraints was met through delivery of planned agendas in advance of the meeting, allowing for personnel to attend only pertinent sections of the meeting, with notes on decisions and action items after all meetings.

Regular stakeholder meetings were also crucial to direct changes to the delivery of model results required to effectively respond to the state’s needs. In particular, it was helpful to set a schedule for regular deliverables and a timeline for incorporation of stakeholder requests. Another key pillar to our success was the use of management tools, such as roles and responsibilities documents and organizational charts. These documents ensured that as the project team morphed and changed, staff remained informed of their role and assigned tasks. It was also crucial to consider early if additional technical staff were needed and what skills they should possess. This is especially important to avoid not only project delays but also staff burnout.

## Conclusions

The physical and societal impacts of the COVID-19 pandemic are immense. Health systems in particular face intense strain to support surging admissions and to avoid capacity and resource saturation. We have seen that the most advanced health system can be stretched beyond capacity, highlighting the importance of forecasting hospital resource usage and needs. To be effective and make informed decisions in a rapidly evolving pandemic environment, key public health stakeholders require up-to-date forecasts that are based on near real-time data. ABMs serve as a vehicle for providing rapid delivery of reliable estimates needed to respond to public health crises, but model development and adaptation in the pandemic environment is not without challenges. Based on our experience, challenges faced in this environment can be distilled to three overarching categories: (1) stakeholder engagement and translation, (2) automation and efficiency, and (3) use of near real-time and constantly evolving data.

### Stakeholder engagement/translation

Above all else, stakeholder engagement and buy-in is the most important element. We worked in collaboration with stakeholders to jointly make decisions throughout the model-building process including in the initial adaptation and in other updates. Stakeholder involvement was central to ensure stakeholder buy-in and the benefits of the model. Stakeholders are relying on the model to make critical decisions and inform public health policy, but even a great model is only useful when the results can be clearly communicated. The adage “a picture is worth a thousand words” comes into play here. ABM visualizations proved to be an exceedingly helpful tool for clearly communicating model functionality to stakeholders even if they did not have a background in modeling.

### Automation and efficiency

Keeping pace with ever-evolving pandemic-related data is another immense challenge. Our project and staff had to be innovative, agile, and adaptable to accommodate the rapid evolution. Automating each step of the process was key to reducing delivery time and ensuring that analysts had sufficient availability and energy to review the validity and quality of weekly deliverables. Automation also reduced the opportunity for error and ensured consistent deliverable quality, week to week.

### Using near-real-time and updated data

Being in the midst of an epidemic does not mean that obtaining access to data will be easy or expedited. In our case, it was the reverse. Many stakeholders were essential to the pandemic response, which severely limited their availability. For this reason, it is essential that the role and importance of specific data sources be communicated early and clearly. Additionally, real-time data are messy, especially when those data rely on numerous reporting systems. If the data received are constantly changing, it is vital that an automated checking system be implemented immediately. Write tests that can catch and identify issues enabling early mitigation in the model pipeline. To be relevant, the model must also adapt as new information becomes available. Avoid misunderstandings and ensure timely updates by developing documentation for system revision, including the process for identifying and vetting parameter estimates and obtaining stakeholder buy-in.

In conclusion, the COVID-19 pandemic exemplifies the importance of anticipating hospital resource needs. Modeling serves as a useful tool for providing leaders with rapid and reliable data needed to better prepare for and respond to such crises. But the use of simulation models in a pandemic environment is not without challenge. By reflecting on these challenges, we illuminate valuable lessons that can inform strategic resource allocation decisions and control of future pandemics. A checklist to guide rapid model development is provided in S1 File.

## Supporting information

S1 File Checklist for rapid model development. (PDF)
