# Supplementary material for: Lessons learned from the rapid development of a statewide simulation model for predicting COVID-19’s impact on healthcare resources and capacity
Source: PLoS One. 2021 Nov 18;16(11):e0260310. doi: 10.1371/journal.pone.0260310 (PMC8601549; doi:10.1371/journal.pone.0260310)
Supplement: S1 File — (PDF) [file pone.0260310.s001.pdf]

1. Modeling
    - Draft a diagram of model processes.
    - Create a list of parameters needed in the model to accompany diagram.
    - Identify preliminary estimates or data sources for parameters.
        i. Note parameters that are particularly crucial.
        ii. Note parameters that should be sampled from distributions for variation.
    - Review diagram and parameter list with stakeholder and fill in gaps.
        i. Particular attention should be paid to the most crucial parameters.
    - Develop prototype version of model.
    - Write tests to catch inconsistencies in real-time data streams.
    - Develop model to use distributions instead of point estimates for key parameters to enable what-if questions and sensitivity analyses.
    - Test model and quantify problems caused by missing/outdated data; share with client to expedite data requests.
    - Develop a model animation to illustrate the model process.
        i. Particularly useful to develop early in modeling process when collaborating with stakeholders who are unfamiliar with ABMs
2. Scaling
    - Estimate computing needs before the project starts and when determining the budget.
        i. Computing needs should consider batch processing and parallel computing opportunities
    - Start building computing infrastructure early.
    - Only use Spot instances to save money if your workloads are fault-tolerant and flexible; otherwise, dedicated instances are worth the extra cost.
    - Automate repetitive tasks that don't require an analyst in the loop.

- Don't automate tasks that aren't well suited for automation (fragile, ever-changing, or requiring an analyst in the loop).

3. Reporting
    - Create a list of outputs from the model.
    - Review list of outputs with client and determine which are most useful for deliverables, how they might be delivered (e.g., Excel, PowerPoint, Tableau, animation), and what visualizations might be used.
    - Create prototype deliverables.
    - Receive feedback from client on prototype deliverables.
        i. Show the deliverables early in the project
        ii. Iterate as necessary on prototypes
    - Upon creation of a final version, consider investing in automation of the deliverable generation process.
        i. Open-source packages from Python are particularly useful for PowerPoint and Excel automation
4. Documentation
    - Use a version control tool like Git, including features like issues, branching, and wikis.
    - Document early and rigorously.
    - Require review of updated documentation as a part of code review.
    - For agent-based models, start writing the ODD early and keep it updated when new features are added.
5. Stakeholder engagement/buy-in (incl. animations)
    - Determine core group of technical team members and stakeholders and decide on a regular meeting schedule.
    - Share agendas before meetings and notes and action items after meetings.

- Use management tools like org charts to manage change in the team over time.
- Set expectations early for deliverable schedule and timeline of incorporation of stakeholder feedback.
- Create a user-friendly visualizations to clearly communicate model behavior with stakeholders.
